# Home-based rehabilitation training with human key point detection for chronic low back pain patients: a randomized controlled trial protocol

**DOI:** 10.1186/s13063-023-07805-z

**Published:** 2023-11-27

**Authors:** Zheng Fuming, Li Zhicheng, Huang Huanjie, Zhang Xinna, Chen Rong, Peng Jiahui, Yang Liming, Chen Xi, Wang Chuhuai

**Affiliations:** 1 Department of Rehabilitation Medicine, The First Affiliated Hospital, Sun Yat-Sen University, Guangzhou, 510080 China; 2 Yinshan Future Health Technology Co., Ltd, Beijing, 100080 China

**Keywords:** Non-specific chronic low back pain, Core stability exercise, Human key point detection, Mobile health

## Abstract

**Background:**

Core stability exercise (CSE) is a globally acknowledged intervention for managing chronic low back pain. However, the sustained adherence of patients with chronic low back pain to CSE can be challenging, mainly due to the absence of supervision and guidance from physical therapists during home-based exercise sessions. Consequently, exercise compliance tends to decline, resulting in suboptimal long-term effectiveness of the intervention. In this trial, our primary aim is to evaluate the potential therapeutic equivalence between home-based rehabilitation training employing key point identification technology and exercise guidance administered in a hospital setting.

**Methods:**

In this trial, we will randomly assign 104 adults with chronic low back pain (CLBP) to either an intervention or control group, with 52 participants in each group. Both interventions will consist of three weekly 0.5-h sessions of core stability exercise (CSE). The intervention group will engage in home rehabilitation training utilizing key identification technology for movement, while the control group will perform supervised exercises in a hospital setting. Outcome assessments will be conducted at 4 weeks and 16 weeks after randomization. The primary outcome measure will be the change in pain intensity based on numeric rating scale (NRS scores) from baseline to 4 weeks. Secondary outcomes will include changes in physical function (measured by the Oswestry Disability Index (ODI)) and lumbar spine mobility as well as activity participation and treatment satisfaction.

**Discussion:**

If home-based rehabilitation method is demonstrated to be non-inferior or even superior to traditional face-to-face exercise guidance, it could significantly advance the adoption of digital medical care and contribute to improving the overall health of the population.

**Trial registration:**

NCT05998434. Registered on 16 August 2023.

## Introduction

### Background and rationale {6a}

Low back pain (LBP) is a significant global public health issue that affects populations across different regions and age groups [1]. Nonspecific chronic low back pain (NCLBP), also referred to as chronic low back pain (CLBP), is characterized by low back pain lasting for more than 3 months without identifiable pathological anatomical factors [2]. The global burden of low back pain is substantial, with an estimated 568.4 million cases reported in 2019, equating to 6972.5 cases per 100,000 individuals [3]. The incidence of low back pain is increasing, and it is projected that by 2050, approximately 843 million individuals worldwide will be affected by this condition due to population growth and aging [4]. With the popularity of smartphones and computers, prolonged sitting and poor posture, such as forward head posture, have become prevalent behaviors among the majority of people [5]. The incidence of spinal pain, particularly neck and lower back pain, continues to increase, leading to an increased rate of sick leave and imposing significant medical and economic burdens on society [6]. In China, the prevalence of chronic pain is reported to be 31.54%, with low back pain being the most prevalent condition at 74.1% [7].

Core stability exercise (CSE) has demonstrated effectiveness in treating chronic low back pain [8–10]. However, frequent visits to the hospital for therapy impose significant time and financial burdens on patients [11]. Traditional home rehabilitation training, lacking supervision and guidance from therapists, often results in suboptimal long-term efficacy of exercise therapy [12]. Therefore, it is essential to develop a convenient and reliable intelligent spinal health AI management system that integrates risk prediction, diagnosis, assessment, treatment, and rehabilitation.

Tele-rehabilitation technology based on mobile health (m-health) has experienced rapid development, especially during the COVID-19 outbreak [13]. This technology enables patients to receive exercise instructions through mobile devices and engage in rehabilitation treatment anytime and anywhere [14]. Our preliminary exploratory study has indicated that core stability training instruction provided remotely has the potential to improve lower back function and reduce pain intensity in patients with chronic low back pain [15]. Research has demonstrated that remote education and exercise guidance via videoconferencing hold promise in achieving therapeutic effects [16]. However, these interventions have not provided patients with objective and accurate supervision and exercise guidance in their homes. Research has shown that unsupervised app-based treatments have not demonstrated significant treatment efficacy and cost-effectiveness [17].

Digital exercise-based interventions have shown great potential in the management of musculoskeletal conditions [18]. The study conducted by Thoma et al. demonstrated the effectiveness of the Kaia application as a multidisciplinary back pain intervention, surpassing a combination of physical therapy and online education, for patients with low back pain [19]. However, it remains unclear whether AI-based intelligent home rehabilitation training will yield comparable therapeutic effects to exercise treatment in a hospital setting. In this study, we will employ human key point detection technology to monitor patients in real time during home rehabilitation training. We will record the duration of patients’ training sessions and assess the quality of their movements, comparing these results with those of traditional on-site exercise guidance.

### Objectives {7}

The objective is as follows: to assess the comparable therapeutic effects of home rehabilitation training using human key point detection technology compared to exercise instruction in a hospital setting.

### Trial design {8}

We are planning to conduct a randomized controlled non-inferiority clinical trial. Eligible participants will be randomly allocated in a 1:1 ratio into either the experimental group or the control group. The experimental group will undergo home rehabilitation training with guidance from human key point detection technology, whereas the control group will receive exercise instruction in a hospital setting. The trial protocol will adhere to the Standard Protocol Items: Recommendations for Interventional Trials (SPIRIT 2013) guidelines for conducting and reporting clinical trials [20].

### Experiment duration

**Fig. 1:**
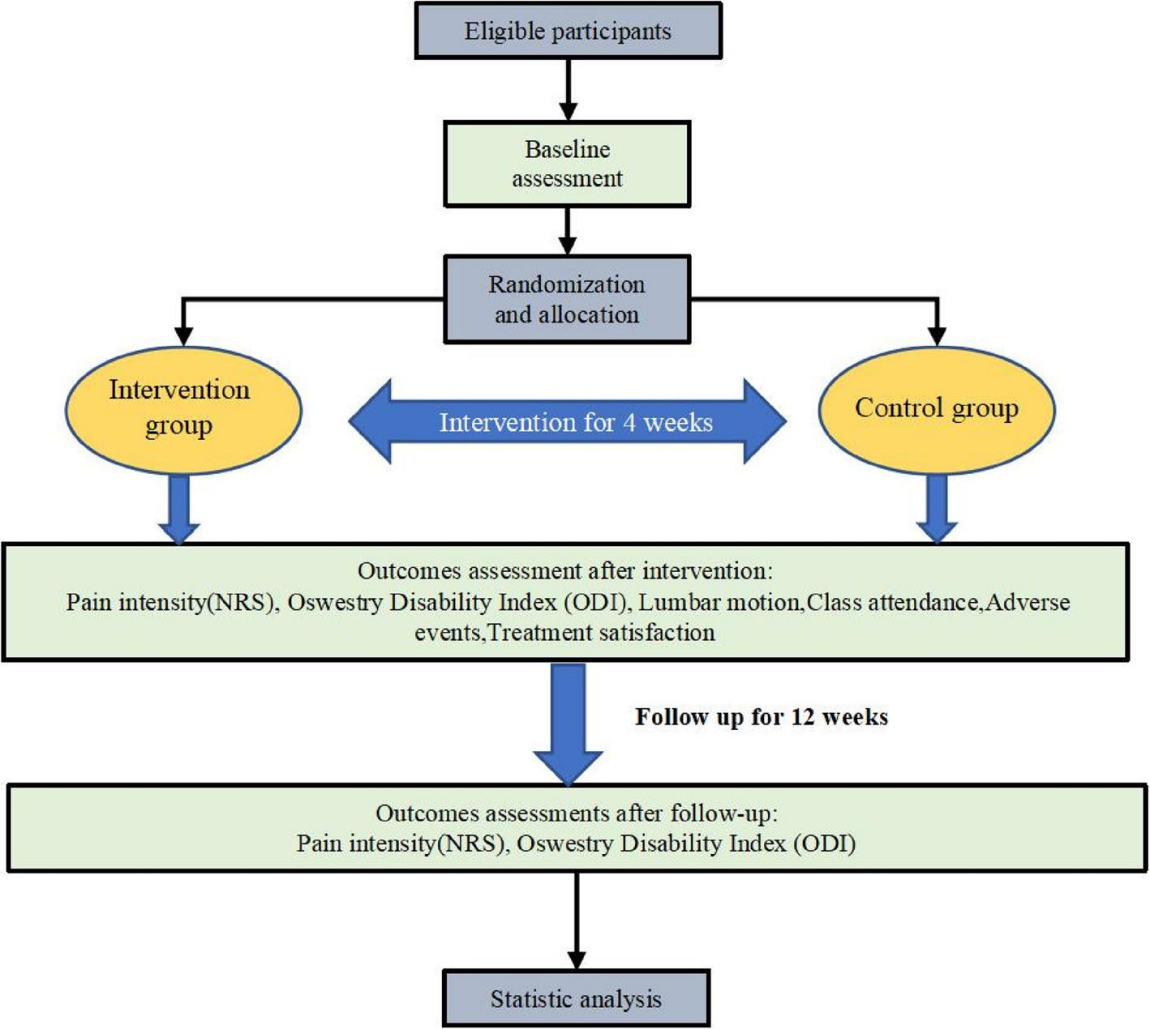
Flow chart

The intervention spanned a total of 4 weeks, with participants engaging in 30-min training sessions three times a week. Clinical follow-up assessments were scheduled at week 4 and week 16. The research project is scheduled to take place from January 2024 to December 2025. The experimental flowchart is shown in the figure (Fig. 1).

## Methods: participants, interventions and outcomes

### Study setting {9}

The RCT is being undertaken in China. The study site is located in the outpatient Department of Rehabilitation of the First Affiliated Hospital of Sun Yat-sen University, and participants in Guangzhou will be mainly recruited for the study.

### Eligibility criteria {10}

Inclusion criteria

- (1) Age between 18 and 60 years.
- (2) Duration of low back pain of at least 12 weeks.
- (3) Strong willingness to participate in the study and signed informed consent.

Exclusion criteria

- (1) Presence of potential “red flag signs” (e.g., unilateral leg pain and numbness consistent with nerve distribution, intermittent claudication, unexplained sudden weight loss, nocturnal lumbar pain, traumatic lumbago).
- (2) NRS score less than 4 points.
- (3) Regular engagement in core stability training in the past 4 weeks.
- (4) Inability to independently complete the Chinese electronic questionnaire.

Exit criteria

- (1) Withdrawal due to sudden personal reasons.
- (2) Withdrawal due to apparent loss of interest in participation.
- (3) Failure to complete the prescribed treatment tasks as required and requesting withdrawal due to unexcused absence.

### Who will take informed consent? {26a}

A study researcher (ZFM) will introduce the trial to potential participants and discuss the trial with them. If the patient agrees, the researcher will obtain a written consent form stating that the patients are willing to participate in the trial.

### Additional consent provisions for collection and use of participant data and biological specimens {26b}

Not applicable, as this study primarily assessed efficacy using subjective scales. This trial does not involve collecting biological specimens for storage.

#### Interventions

### Explanation for the choice of comparators {6b}

Face-to-face core stability training guidance is a traditional and proven treatment method. However, it may not be feasible for individuals residing in remote areas or with busy schedules. To verify the effectiveness of home-based rehabilitation, we selected traditional face-to-face exercise guidance as the control group.

#### Intervention description {11a}

### Intervention group

**Fig. 2:**
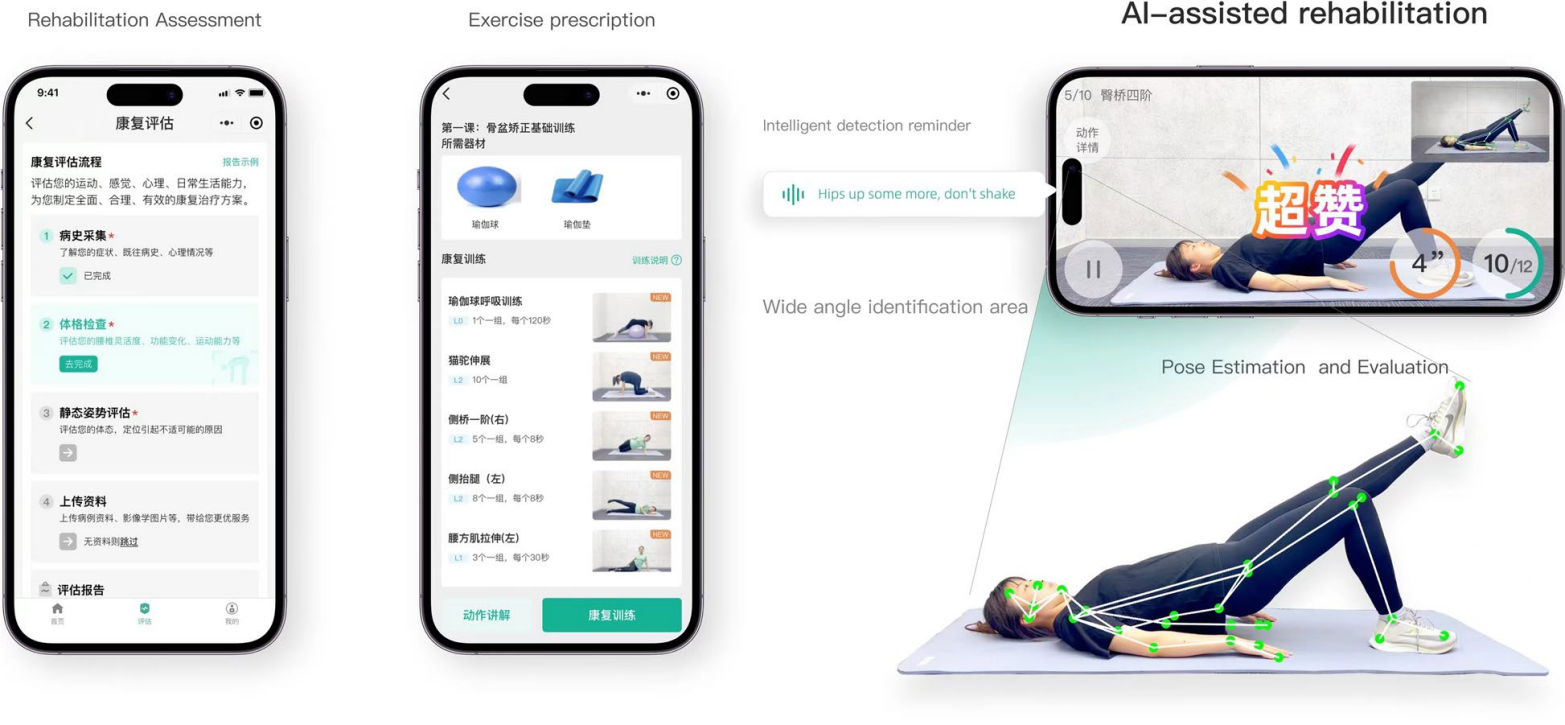
Example of Yinshan mini program

The participants randomly assigned to the intervention group will receive home-based exercise training with human key point detection. They will be instructed to complete a minimum of three sports training sessions per week at their preferred time and location. The exercise prescription will be formulated based on previous comprehensive studies [21–23], encompassing exercises targeting strength, balance, flexibility, and mobility. Human key point detection technology accurately estimates 25 key points of the human body in pictures or videos using visual detection. These key points include the left and right elbow, left and right wrist, left and right shoulder, head, neck, left and right ankle, left and right knee, and left and right hip, among others. The technology allows for accurate estimation of standing, sitting, and moving postures in various scenes, facilitating the detection and recognition of action postures. Yinshan Future Health Technology (Beijing) independently developed this human body key point detection technology, which includes self-assessment and intelligent rehabilitation training. Participants receive treatment and evaluation through the WeChat mini-program. For autonomous assessment, participants can report their medical history and independently measure their spinal range of motion following the provided prompts. The data is automatically stored for therapists and physicians to review (see Fig. 2 for a specific demonstration).

### Control group

The participants randomly assigned to the control group will receive traditional face-to-face exercise instruction. An experienced therapist (10 years of experience) will be arranged to provide the subjects with 20–30 min of exercise instruction three times a week for 4 weeks [24]. The training sessions were scheduled to start at 4 p.m. on Monday, Wednesday, and Friday of each week. The exercise training content selected for the control group will be consistent with that of the intervention group.

### Criteria for discontinuing or modifying allocated interventions {11b}

1. *Medical safety concerns*: If a participant experiences any adverse events or medical complications that are directly related to the allocated exercise program, discontinuation or modification should be considered. This could include issues like musculoskeletal injuries, cardiovascular symptoms, or other medical conditions exacerbated by exercise.
2. *Non-adherence*: If a participant consistently fails to adhere to the prescribed exercise regimen, it might be necessary to modify the program to better suit their schedule, preferences, or physical capabilities. Non-adherence can affect the efficacy of the intervention and overall study outcomes.
3. *Technology issues*: Given the remote nature of the intervention, technical issues may arise that hinder the participant’s ability to engage effectively. In such cases, modifications to the technology setup or the exercise routine may be required.

### Strategies to improve adherence to interventions {11c}

1. *Personalized planning and goal setting*: Developing individualized rehabilitation plans based on participants’ unique characteristics and objectives enhances intervention adherence. Aligning rehabilitation goals with participants’ expectations and realistic capabilities fosters engagement and persistence. Tailored plans bolster participant motivation, increasing the likelihood of sustained intervention adherence.
2. *Regular tracking and feedback*: Establishing systematic tracking mechanisms to monitor participants’ progress and achievements is imperative. Providing consistent feedback assists participants in recognizing their efforts and advancements. Positive reinforcement fosters affirmative behavior and aids participants in maintaining intervention adherence.
3. *Mobile applications and reminder systems*: Leveraging mobile applications and reminder systems delivers timely prompts and guidance to participants. Mobile applications may encompass training regimens, video tutorials, and progress monitoring. Reminder systems, through short messaging service or emails, prompt participants to fulfill their intervention tasks. These technological tools heighten participant engagement with the intervention, mitigating forgetfulness and thus amplifying adherence.

### Relevant concomitant care permitted or prohibited during the trial {11d}

1. *Permitted concomitant care*: Participants are permitted to continue their routine medical care and consultations with their primary healthcare providers. Concomitant medications, advised exercises, and non-invasive interventions unrelated to the trial are allowed. These should be documented and reported to provide a holistic view of participants’ overall care.
2. *Prohibited concomitant care*: During the trial period, participants are advised against engaging in any new therapeutic interventions, whether conventional or complementary, for chronic low back pain. This includes invasive procedures, surgical interventions, and experimental treatments targeting the same condition. Participants are encouraged to strictly adhere to the trial intervention and to refrain from initiating new concomitant care approaches without prior approval from the study investigators.

### Provisions for post-trial care {30}

If participants are injured as a result of this study, they may receive free treatment and/or compensation in accordance with Chinese law in case of injury in connection with this clinical study.

#### Outcomes {12}

### Primary outcome

The primary outcome is the mean pain intensity over the previous week assessed using an 11-point numeric rating scale (NRS;0 = no pain, 10 = unbearable pain) at 16 weeks after randomization. A higher score indicates higher pain intensity [25]. The between-group minimal clinically important difference (MCID) is 1.0 point on the NRS scale [26]. The within-group MCID is a 30% change from baseline [27].

#### Secondary outcomes

1. The disability and will be assessed using the Oswestry Disability Index (ODI). The ODI evaluated disability across 10 domains, encompassing pain intensity and functional abilities related to personal care, lifting, walking, sitting, standing, sleeping, sexual activities, social life, and traveling. Each domain was rated on a 6-point scale ranging from 0 to 5. A score of 0 indicated the highest level of functioning, while a score of 50 indicated total disability [28].
2. The 36-Item Short Form Health Survey (SF-36), a widely used tool, assesses health-related quality of life and overall well-being across diverse populations. Developed by Wareldman and Ware in the early 1980s, it evaluates an individual’s physical, mental, and social health experiences across eight domains, including physical functioning and mental health. With 36 questions, responses are scored from 0 to 100, reflecting higher scores for better health status. Its brevity, versatility, and comprehensive health dimension coverage are notable advantages. The SF-36 finds applications in clinical practice, epidemiology, and health outcome studies, aiding in diagnosis, treatment, and policy decisions. Its widespread use allows for inter-study comparisons and meta-analyses, underpinned by robust psychometric properties, making it an invaluable tool for assessing health-related quality of life across diverse contexts [29].
3. We will measure the angles of forward flexion, extension, and lateral bending in patients with CLBP using measurement tools within the Yinshan system.

In the control group, participants will be required to sign in and record their attendance. The intervention group utilized a WeChat mini program system to document treatment frequency and quality for the participants. This study excludes the use of drugs or invasive procedures, thereby minimizing the potential for adverse reactions. However, it is important to acknowledge that inadequate warm-up, excessive exercise intensity, or improper technique during exercises may exacerbate pain or cause muscle strain and fatigue. To promptly address any potential adverse reactions, participants will receive instructions to immediately contact us in case of physical discomfort. Patient satisfaction will be assessed using the Patient Satisfaction Questionnaire [30], a straightforward survey that measures treatment satisfaction on a scale of 1 to 5, with the following criteria: 1 = satisfied, 2 = somewhat satisfied, 3 = neither satisfied nor dissatisfied, 4 = somewhat dissatisfied, and 5 = dissatisfied.

### Participant timeline {13}

The participant timeline is presented in Table 1.

**Table 1:**
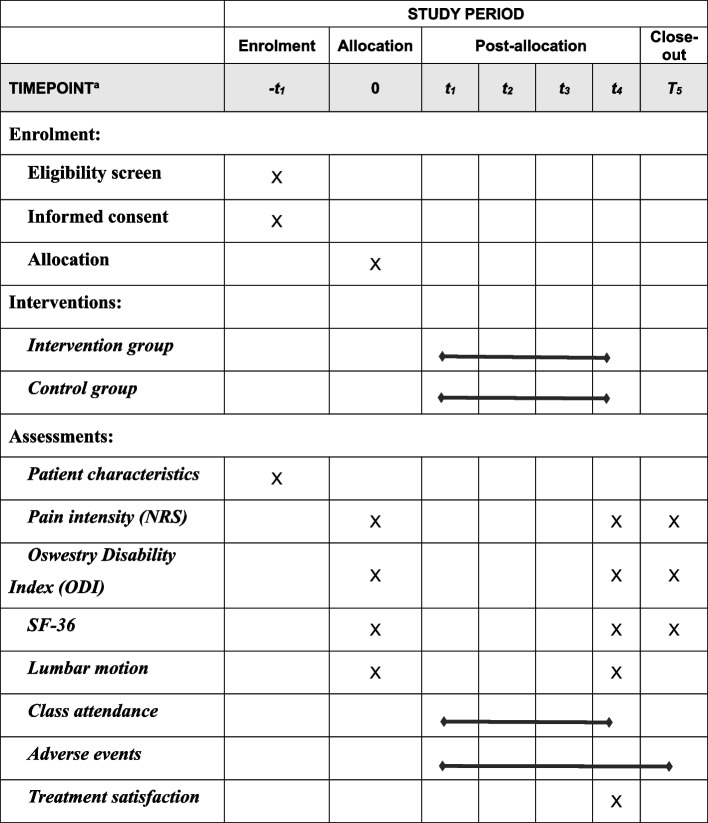
Content for the schedule of enrolment, interventions and assessments

^a^*t*_*1*_, week 1; *t*_*2*_, week 2; *t*_*3*_, week 3; *t*_*4*_, week 4; *T*_*5*_, week 16

### Sample size {14}

With a non-inferiority design, the intervention group and control group had a 1:1 ratio of participants. In March 2023, we conducted a pilot study in the outpatient department of the First Affiliated Hospital of Sun Yat-sen University and collected a total of 21 subjects, including 15 in the intervention group and 6 in the control group, for a 4-week intervention. They were followed up 3 months later. The result yielded mean decreases of − 1.6 (SD = 2.0) for the intervention group and − 1.9 (SD = 1.9) for the control group on the NRS. With a significance level (*α*) of 0.05 and a power (1-*β*) of 0.9, considering a non-inferiority margin of − 1 and a difference (*δ*) of 0.3 in NRS decrease between the intervention and control groups, the PASS software (version 21) estimated that each group required 41 participants. Assuming a dropout rate of 20%, a total of at least 102 participants were needed for both groups.

### Recruitment {15}

Recruitment will be advertised in notices posted in the clinic and on a social network platform (WeChat). The main inclusion and exclusion criteria will be included in our registration questionnaire. Possible eligible participants will be invited to come to the hospital for a physical examination and baseline assessment. Eligible participants will be assigned a number according to the sequence of their arrival at the hospital and then to the research assistant for random grouping. Participants will be informed of the time of treatment after the baseline assessment, so that they can arrange their schedule. Treatment content will be announced 3 days before the start of the intervention.

#### Assignment of interventions: allocation

### Sequence generation {16a}

For the randomization, a simple block randomization process was conceived and will be implemented by a trial assistant. After eligibility is confirmed, every participant will be assigned a unique number as an identifier. Sequence generation will be achieved using the IBM Statistical Package for Social Sciences (SPSS) version 23 software and stratified with a 1:1 allocation. The randomization list reports a progressive randomization number for randomized participants (from 1 to 20), and the treatment (intervention or control) will be assigned to the subject in either group.

### Concealment mechanism {16b}

The randomization tables will be generated by a research assistant who will not participate in the entire participant recruitment and treatment process and will be kept by another researcher who will not participate in the participant recruitment. The randomization tables will be published within 1–2 days of the intervention. This ensures that the researchers, therapists, and subjects in charge of recruitment do not know about the grouping before the intervention begins.

### Implementation {16c}

The trial assistant, who will not be involved in the entire treatment procedure, will generate and maintain the allocation sequence. The principal investigator will enroll and assign the participants to the groups according to their unique numbers. Once allocated, the participant will not be allowed to change their group.

#### Assignment of interventions: blinding

### Who will be blinded {17a}

It is not possible to blind the participants and the researcher due to the nature of the experiment’s design.

### Procedure for unblinding if needed {17b}

Not applicable as no blinding was used in this trial.

#### Data collection and management

### Plans for assessment and collection of outcomes {18a}

Data collection will be facilitated through an electronic questionnaire, with automatic storage in a network disk database (https://www.wjx.cn/newwjx/manage/myquestionnaires.aspx). This database will be subject to collaborative management by the research team. Subsequent data analysis will be conducted by a statistical analyst, ensuring blinding to both group allocations and intervention protocols.

### Plans to promote participant retention and complete follow-up {18b}

1. *Clear communication and participant engagement*: Establish open and transparent communication channels with participants from the beginning of the study. Clearly explain the study’s importance, goals, and expected outcomes. Maintain regular contact through WeChat to keep participants engaged and informed about the study’s progress. Emphasize the significance of their contribution to foster a sense of ownership and commitment.
2. *Incentives and acknowledgments*: Offer appropriate incentives as a token of appreciation for participants’ commitment to the study. We plan to give the subject a lumbar pad worth about 50 yuan after completing the 4-week treatment. Recognizing their dedication can enhance their motivation to stay involved.

### Data management {19}

1. *Data validation and quality control*: Implement rigorous data validation and quality control procedures. This includes real-time validation checks during data entry to identify errors, inconsistencies, or missing values. Conduct routine data audits to ensure accuracy and integrity. Develop a clear protocol for resolving discrepancies and data outliers. Maintaining high data quality enhances the reliability and validity of research outcomes.
2. *Documentation and version control*: Maintain comprehensive documentation of the data management process. Document data collection procedures, data cleaning processes, and any changes made to the dataset. Establish version control to track dataset modifications over time. This documentation not only ensures transparency but also assists in reproducibility and future analyses.

### Confidentiality {27}

Prioritize data security and privacy throughout the data management process. Implement encryption measures to protect sensitive participant information during transmission and storage. Each participant will be assigned an identification number to be stored in place of their real name. Establish strict access controls to limit data access to authorized personnel only. Regularly update and patch software to mitigate vulnerabilities.

### Plans for collection, laboratory evaluation and storage of biological specimens for genetic or molecular analysis in this trial/future use {33}

This trial does not involve the collection of biological samples for genetic or molecular analysis.

## Statistical methods

### Statistical methods for primary and secondary outcomes {20a}

Statistical analysis was conducted according to intention-to-treat (ITT). The chi-square test was used to analyze categorical variables when comparing baseline demographic and clinical characteristics; For continuous variables, we first performed exploratory data analysis and Shapiro–Wilk tests to determine the normality of our data distribution, using independent *t* tests when the results conform to a normal curve and non-parametric rank sum tests when they do not.

To analyze the data, we will employ repeated measures analysis of variance (RM-ANOVA) as the statistical method. Our primary interest lies in comparing the changes over time between the intervention and control groups at baseline, 4 weeks, and 16 weeks. We will utilize repeated measures ANOVA to examine the main effects between the two groups while considering the factors of time and group. As part of the analysis, multiple comparisons will be performed, including comparisons between baseline and 4 weeks, baseline and 16 weeks, and 4 weeks and 16 weeks. These comparisons will help identify significant differences across different time points. To assess statistical significance, appropriate statistical indicators such as *F*-values and *p*-values will be utilized. In case of significant differences, post hoc multiple comparison analyses will be conducted to determine specific time points of significance. Finally, descriptive statistics and appropriate graphical representations will be employed to illustrate the overall trends and changes in the data. Statistical analyses were done using the SPSS software for Windows, version 23.

### Interim analyses {21b}

1. *Timing of interim analyses*: Interim analyses will be conducted at pre-defined time points during the trial. These points will be carefully selected to ensure meaningful insights while minimizing the risk of bias. Interim analyses are planned to occur after approximately 50% of participants have completed the trial and before the final analysis.
2. *Statistical methods*: Interim analyses will be performed using appropriate statistical methods to ensure robustness and validity. An independent statistical analyst not involved in the day-to-day conduct of the trial will perform the analyses. To maintain statistical integrity, these analyses will utilize adjusted significance thresholds to account for multiplicity issues arising from multiple looks at the data.
3. *Stopping guidelines*: Well-defined stopping guidelines will be established in advance to guide decision-making based on interim results. Stopping criteria will be based on pre-specified thresholds for safety or efficacy, as determined through statistical methods such as Bayesian or frequentist approaches. The researchers (CX and WCH) will review the interim findings and make recommendations regarding study continuation or modification.

### Methods for additional analyses (e.g., subgroup analyses) {20b}

We plan to classify the severity of each group of patients into severe pain and moderate pain according to the NRS and analyze whether there are differences in changes between them.

### Methods in analysis to handle protocol non-adherence and any statistical methods to handle missing data {20c}

For continuous outcomes, such as numeric rating scale (NRS) scores, a multiple imputation approach will be utilized to address missing data. Imputation will be performed using covariates and auxiliary variables that are pertinent to the analysis. The imputed datasets will be subject to analysis, and the outcomes will be aggregated to ensure unbiased estimations and reliable inferences. To enhance the robustness of findings in the presence of missing data, sensitivity analyses will be conducted. These analyses will evaluate the resilience of results to diverse assumptions concerning missing data. Variation in imputation methodologies or mechanisms will be explored to assess their potential impact on the derived conclusions. By incorporating sensitivity analyses, the credibility of outcomes will be fortified across a spectrum of scenarios.

### Plans to give access to the full protocol participant-level data and statistical code {31c}

In accordance with the data sharing statement, we commit to providing a fully deidentified dataset to a suitable data repository for the purpose of sharing. This will be done within 3 years following the completion of the 1-year post-randomization interviews.

#### Oversight and monitoring

### Composition of the coordinating center and trial steering committee {5d}

The researchers and all aspects of the study will be supervised by the ethics committee of the First Affiliated Hospital of Sun Yat-sen University, who will ensure that the study adheres to ethical principles and protects the patient’s health and dignity.

### Composition of the data monitoring committee, its role and reporting structure {21a}

A data monitoring committee (DMC) is not needed, because our trial is short duration and with known minimal risks. But the researchers (CX and WCH) will regularly analyze the data to make adjustments.

### Adverse event reporting and harms {22}

Adverse events related to the remote rehabilitation, primarily musculoskeletal strains from exercise, will be diligently reported and managed. Participants will be encouraged to maintain a daily training diary to monitor their physical conditions. This proactive approach enables swift identification and mitigation of any potential harms, ensuring participant safety during the intervention.

### Frequency and plans for auditing trial conduct {23}

Our study incorporates a scheduled audit every 4 weeks. The audit will encompass diverse processes, including participant enrollment, informed consent, eligibility assessment, and allocation to study groups. Additionally, it will scrutinize adherence to trial interventions, participant safety measures, reporting of adverse events, and the thoroughness, precision, and punctuality of data collection. This routine auditing reinforces the methodological rigor and ethical compliance of the study.

### Plans for communicating important protocol amendments to relevant parties (e.g., trial participants, ethical committees) {25}

Any alterations to the protocol that could influence the study’s implementation, potential patient benefits, or impact patient safety will necessitate a formal protocol amendment. This encompasses modifications to study objectives, design, patient demographics, sample sizes, procedures, or substantial administrative aspects. All proposed changes will be submitted for approval to the ethics committee of the First Affiliated Hospital of Sun Yat-sen University. Implementation of the revised protocol will proceed only after obtaining the requisite ethical approval.

### Dissemination plans {31a}

The study results will be released to the participants, healthcare professionals, the public, and other relevant groups via publication.

## Discussion

The application of human key point detection technology holds promising potential in advancing home-based rehabilitation for individuals with chronic lower back pain. By leveraging this technology, patients can receive personalized guidance and real-time feedback on exercise techniques and postures, enhancing the effectiveness of their rehabilitation efforts.

Human key point detection’s ability to accurately track and analyze body movements offers several advantages [31]. It enables objective assessment of exercise performance, ensuring that patients engage in correct and safe movements. This technology’s interactive nature fosters engagement and adherence by providing patients with immediate visual cues for adjustments. Moreover, it empowers patients with a sense of autonomy over their rehabilitation process, which can contribute to improved motivation and long-term commitment.

Additionally, incorporating human key point detection into home-based rehabilitation facilitates remote monitoring by healthcare professionals [17]. Clinicians can remotely assess patients’ exercise compliance, identify potential issues, and make timely adjustments to treatment plans. This real-time communication strengthens the patient-provider relationship, enhances patient outcomes, and reduces the need for frequent in-person appointments.

However, challenges such as data privacy, technological accessibility, and the need for user-friendly interfaces warrant consideration. Ensuring secure data transmission and storage is imperative to protect patient information. Addressing technological barriers among diverse patient populations will be essential for equitable implementation.

In conclusion, the integration of human key point detection technology into home-based rehabilitation for chronic lower back pain patients holds the potential to revolutionize personalized care delivery. Its real-time guidance, interactive features, and remote monitoring capabilities align with the growing demand for patient-centric, technology-assisted healthcare solutions. Further research and collaboration between technological developers and healthcare professionals will be pivotal in realizing the full benefits of this innovative approach.

## Trial status

The project is currently in its preliminary research phase, with plans to commence participant recruitment in January 2025. The trial is anticipated to conclude, including all follow-up assessments, by December 2026.

## Data Availability

Data for the study can be made available upon request. Interested researchers should contact Dr. Zheng at zhengfm7@mail2.sysu.edu.cn.

## Abbreviations

CLBP: Chronic low back pain
CSE: Core Stability Exercise
NRS: Numeric Rating Scale
ODI: The Oswestry Disability Index
SF-36: 36-Item Short Form Health Survey
MCID: Minimal clinically important difference
m-health: Mobile health
ITT: Intention-to-treat
RM-ANOVA: Repeated measures analysis of variance
